# Draft Genome Sequence of *Agrobacterium* sp. Strain R89-1, a Morphine Alkaloid-Biotransforming Bacterium

**DOI:** 10.1128/genomeA.00196-16

**Published:** 2016-04-07

**Authors:** Jiří Zahradník, Eva Kyslíková, Pavel Kyslík

**Affiliations:** a Laboratory of Enzyme Technology, Institute of Microbiology CAS, v.v.i., Prague, Czech Republic; b Department of Genetics and Microbiology, Faculty of Science, Charles University Prague, Prague, Czech Republic; c Department of Biochemistry, Faculty of Science, Charles University Prague, Prague, Czech Republic

## Abstract

*Agrobacterium* sp. strain R89-1 isolated from composted wastes of *Papaver somniferum* can effectively biotransform codeine/morphine into 14-OH-derivatives. Here, we present a 4.7-Mb assembly of the R89-1 strain genome. The draft shows that the strain R89-1 represents a distinct phylogenetic lineage within the genus *Agrobacterium.*

## GENOME ANNOUNCEMENT

*Agrobacterium* is a genus of Gram-negative alphaproteobacteria predominantly inhabiting rhizosphere or soil. They usually induce plant tumors by transferring genes from an indigenous Ti plasmid to plant genomes.

The *Agrobacterium* sp. strain R89-1 was originally isolated from composted poppy seed wastes (*Papaver somniferum*) in the central Bohemia region (Neratovice, Agro Býškovice) as a nonphytopathogenic strain capable of codeine 14-OH biotransformation (1). The strain was deposited in the Czech Collection of Microorganisms (CCM) with the number CCM 7949. Researchers are focusing on the opiate alkaloid’s biotransformation and degradatory pathway due to the high potential of *de novo* synthesis of morphine by yeast (2, 3). On the other hand, morphine and its derivatives are widely used as analgesics and antitussives, and their occurrence and accumulation as serious pollutants in the environment has been reported (4) together with their biological effects (5).

The total genomic DNA was isolated with a bacterial genomic DNA isolation kit (Norgen, Canada) according to the manufacturer’s protocol. The genome sequence was determined using an Illumina HiSeq 2000 sequencing platform (Illumina) with the paired-end approach of the Beijing Genomics Institute. A total of 5,778,196 reads comprising 718 Mb of clean data (722 Mbp) were assembled using SOAPdenovo (version 2.04) and SOAPaligner (version 2.21) was used to align reads to the reference sequence of *Agrobacterium* sp. H13-3. Comparing with reference *Agrobacterium* sp. H13-3, we found that in corresponding regions *Agrobacterium* sp. R89-1 contains 31,333 single nucleotide polymorphisms (SNPs) including 23,197 synonymous mutations and 6,844 nonsynonymous mutations. The annotation pipeline BASys (6) predicted 5,105 coding genes with more than a third of unknown or unpredicted/unpredictable functions (34%). The RNAmmer prediction server identified 3 rRNAs and 45 tRNAs in the genome (7). The presence of a tumor-inducing plasmid was not identified by BLAST search. The draft genome sequence of *Agrobacterium* sp. R89-1 has an overall G+C content of 56.26%. It consists of 4,750,408 bp in 30 scaffolds. The gene encoding protelomerase (*telA*) was detected and therefore we suggested the presence of simultaneous linear and circular chromosomes in the genome (8). Multilocus sequence analysis (MLSA) based on *atpD*, *glnA*, *gyrB*, *recA*, and average nucleotide identity (ANI) values significantly showed that the strain R89-1 represents a distinct phylogenetic lineage within the genus *Agrobacterium*. The nearest 16S rDNA homologue with 8 mutations in the gene belongs to *Agrobacterium* sp. NCPPB 1650 (GenBank accession no. D14506.1). Moreover, the *recA* allele analysis showed an identity value of only 93% with the homologue NCPPB 1650 (9), therefore, the strain R89-1 forms a specific branch clearly different from the genomic species *Agrobacterium rubi* with an ANI value of 81.5 [72.3] computed by JspeciesWS software (10). The taxonomy of *Agrobacteria* is still under revision (11), therefore we classified the strain only as *Agrobacterium* sp.

The genome draft of *Agrobacterium* sp. R89-1 reported by us may serve as a genomic view of the opiate alkaloid degradation pathway/catabolism, to elucidate physiological responses of the bacterium to opiate stress, and to extend our knowledge of the *Agrobacterium* sp. cluster.

### Nucleotide sequence accession numbers.

Results obtained under this whole-genome shotgun project have been deposited at DDBJ/EMBL/GenBank under the accession no. LNUW00000000. The version described here is version LNUW01000000.
